# Effects of resistance and balance exercises for athletic ability and quality of life in people with osteoporotic vertebral fracture: Systematic review and meta-analysis of randomized control trials

**DOI:** 10.3389/fmed.2023.1135063

**Published:** 2023-03-09

**Authors:** Xuefei Li, Wenhua Chen, Qian Chen, Fangfang Li, Chen Chen, Pan Li, Fangyu Li, Suxia Guo, Pinghua Chen, Weina Yuan, Dan Liu, Shiyun Wang, Zhijun Hu

**Affiliations:** ^1^Longhua Clinical Medical College of Shanghai University of Traditional Chinese Medicine, Shanghai, China; ^2^School of Pharmaceutical Sciences, Zhejiang Chinese Medical University, Hangzhou, China; ^3^Longhua Hospital Affiliated to Shanghai University of Traditional Chinese Medicine, Shanghai, China; ^4^Shanghai Seventh People's Hospital, Shanghai, China

**Keywords:** resistance and balance exercise, osteoporotic vertebral fracture, functional status and balance, randomized control trials, systematic review and meta-analysis

## Abstract

**Purpose:**

This study aimed to use meta-analysis to determine the impact of resistance and balance training on athletic ability and quality of life for patients with osteoporotic vertebral fracture (OVF).

**Methods:**

This study followed the Preferred Reporting Items for Systematic Review and Meta-Analysis (PRISMA) criteria for systematic reviews and meta-analyzes. The PubMed, Web of science, Cochrane, Embase, and CNKI databases were searched for randomized controlled trials (RCTs) up to September 2022. The search strategy was related to the intervention measures, population, and results, and was structured around the search terms: “Exercise,” “Osteoporotic vertebral fracture,” and “activities of function.” Two reviewers strictly implemented the inclusion and exclusion criteria. Subgroup analyzes of age and training duration were performed for the main outcomes.

**Results:**

We included 12 RCTs (*n* = 1,289) of resistance and balance training in patients with OVF. Compared with controls, the intervention group showed improvements on the Quality of Life Questionnaire issued by the European Foundation for Osteoporosis, visual analog pain scale, Timed Up and Go, falls efficacy scale international (FES-I), kyphosis, and functional reach. On subgroup analysis, the effect was more significant when training continued >10 weeks.

**Conclusion:**

Resistance and balance exercise training improved function and balance, and reduced fall risk in patients with OVF. We recommend resistance and balance training for at least 10 weeks. Future multicenter, large sample trials are needed for more reliable conclusions.

## Introduction

Osteoporosis is a metabolic bone disease characterized by decreased bone mass and deterioration of bone tissue microstructure that is common around the world (1). Osteoporotic vertebral fracture (OVF) is one of the most common consequences of osteoporosis (2). A cross-sectional study of the Chinese mainland found that the prevalence of osteoporosis in men and women over 40 years old was 5.0% and 26.0%, respectively, and that OVF occurred in 10.5% of men and 9.7% of women. Although this incidence of osteoporosis and fracture in China is very high, few patients have received osteoporosis treatment; thus, it has been considered a “silent 21st century epidemic” (3, 4). However, its treatment cost remains huge. In the United States, the high medical resource utilization rate and medical costs of OVF have far exceeded the costs of stroke, myocardial infarction, and breast cancer (5). OVF can result in loss of height, acute and chronic pain, impaired ambulation/balance, decreased quality of life, and shortened life span (6). More importantly, OVF can also lead to increased future re-fracture risk (7, 8). Falls have been considered the primary risk of fracture in patients with OVF (9). Therefore, reducing falls and improving body balance in patients with OVF are considered important measures to reduce re-fracture.

Exercise can delay the negative effects of chronic aging diseases on the body (10). For osteoporosis, exercise is a safe and low-cost non-medication intervention (11). Exercise can reduce the estimated loss by maintaining the cortical and trabecular bone density. It can also improve patient function, including exercise ability and balance, and back muscle strength (12, 13). Therefore, it is strongly recommended that patients with osteoporosis take part in exercise, especially balance and resistance strength training (14). One study found that resistance and balance exercises significantly enhanced lumbar muscle strength, reduce bone loss, and decreased lumbar fracture incidence in postmenopausal women (15). Is exercise beneficial for patients with OVF? A literature search revealed one previously published systematic review on the impact of exercise on patients with OVF, which was unable to determine clear benefits of exercise in people with spinal fractures (16). However, the investigation was assessing simple exercise rather than a specific type of training. In recent years, resistance and balance training have been gradually applied as a composite exercise program to intervene for OVF. Nevertheless, a specific relationship of resistance and balance exercise with OVF has not been previously quantified. It is essential to clarify the specific therapeutic effects (e.g., enhancing motor and balance function, reducing back pain and fear of falling) of resistance and balance training for patients with OVF, because it could impact their rapid rehabilitation significantly. Therefore, we conducted this systematic review and meta-analysis evaluating the use of resistance and balance exercise training for OVF.

### Information sources and search strategy

The referenced data was searched in the following electronic databases: PubMed, Web of Science, Cochrane, Embase and CNKI. We systematically searched the above databases for articles published up until September 17, 2022, without language restrictions. The search strategy was related to intervention measures, population, and results, and was structured around the search terms: “Exercise,” “Osteoporotic vertebral fracture” and “Functional activities.” Keywords and their synonyms were used to improve search sensitivity: (“Exercises” OR “Activities, Physical” OR “Activity, Physical” OR “Physical Activities” OR “Exercise, Physical” OR “Exercises, Physical” OR “Physical Exercise” OR “Physical Exercises” OR “Exercise Training” OR “Exercise Training” OR “Training, Exercise” OR “Training, Exercise”) AND (“Osteoporotic fracture” OR “Fractures, Osteoporotic” OR “Fracture, Osteoporotic”) AND (“Fracture, Spinal” OR “Fractures, Spinal” OR “Spinal Fracture”) AND (“Function” OR “Activities of daily living” OR “Functioning”). In PubMed, search results were limited to “randomized controlled trials.” Search strategy in Supplementary Document. The first author (LXF) screened studies by title and abstracts according to the inclusion and exclusion criteria. In addition, a manual search in the references and abstracts of all included articles and previous relevant systematic reviews and meta-analyzes was carried out. The standards of the Preferred Reporting Items for Systematic Review and Meta-Analysis (PRISMA) guided this systematic review and meta-analysis (17).

### Inclusion criteria

The participants, intervention, comparison, outcome, time, and study design (PICOTS) criteria were considered to determine the study inclusion criteria: (1) The participants had been diagnosed with osteoporosis and suffered at least one vertebral fracture, verified by DXA-based vertebral fracture assessment or X-ray by medical doctors in a clinical setting, (2) The intervention was standardized progressive exercise therapy, especially resistance and balance training, (3) The control group maintained their previous level of daily and physical activities, (4) The outcomes were patients’ balance, mobility, and health-related quality of life, using measures including the “Quality of Life Questionnaire issued by the European Foundation for Osteoporosis” (QUALEFFO-41), Timed Up and Go (TUG), walking speed, VAS (visual analog pain scale), kyphosis, time-loaded standing, etc., and (5). The study design was RCTs published in authoritative journals.

### Exclusion criteria

We excluded studies with the following characteristics: (1) Full text and/or data inaccessible, (2) Participants with other bone metabolic diseases (diabetes, thyroid dysfunction), and (3) Patients with cancer for chemo and/or radiotherapy.

### Data extraction

Two researchers (CWH, CQ) independently extracted data after reading the full text, and the third investigator (LFF) solved any disagreement. The collected information included the first author’s name, publication year, participant characteristics (mean age and gender), sample size, characteristics of exercise intervention (training frequency and intervention duration), risk assessment, and outcome characteristics.

### Outcome measures

In this systematic review and meta-analysis, the primary outcomes were scores of QUALEFFO-41, VAS, and functional reach test (FR) assessments. Secondary measures were scores of the TUG and Falls Efficacy Scale International (FES-I) and measurements of kyphosis.

### Study quality assessment

Two researchers (CC, LP) independently used Cochrane’s collaborative tools (risk of bias) to assess the methodological quality of every RCT. Disagreements were resolved through discussions with the third assessor (LFY). The risk of bias assessment includes random sequence generation, allocation concealment, participants and personnel blinding, outcome assessment blinding, incomplete outcome data, selective reporting, and other bias. All standards were equally estimated with “low,” “unclear,” and “high” risk levels.

### Data synthesis

All analyzes were carried out using Review Manager 5.3 (Nordic Cochrane Center, Copenhagen, Denmark). The extracted results data is completed using changes in the mean and standard deviation (SD) values. Subtract the mean value before the intervention from the mean value after the intervention and calculate the standard deviation of the change according to the number of subjects in the study group combined with the value of group *p* or 95% confidence interval (the changes of mean value and standard deviation are not reported). The χ^2^-test and *I*^2^-value were used to evaluate the heterogeneity of individual research results. The fixed effect model was used when *I*^2^ was less than 50%, and the random effect model was used when *I*^2^ was more than 50%. In addition, subgroup analysis was used to identify potential causes of heterogeneity.

### Subgroup analysis and exploring heterogeneity

In cases of heterogeneity, we expect the following subgroup analyzes (*a priori*): patient’s age (less than or over 70 years) and duration of exercise intervention (less than or over 10 weeks). We planned to use a funnel chart to evaluate for publication bias.

## Results

### Study selection

**Figure 1:**
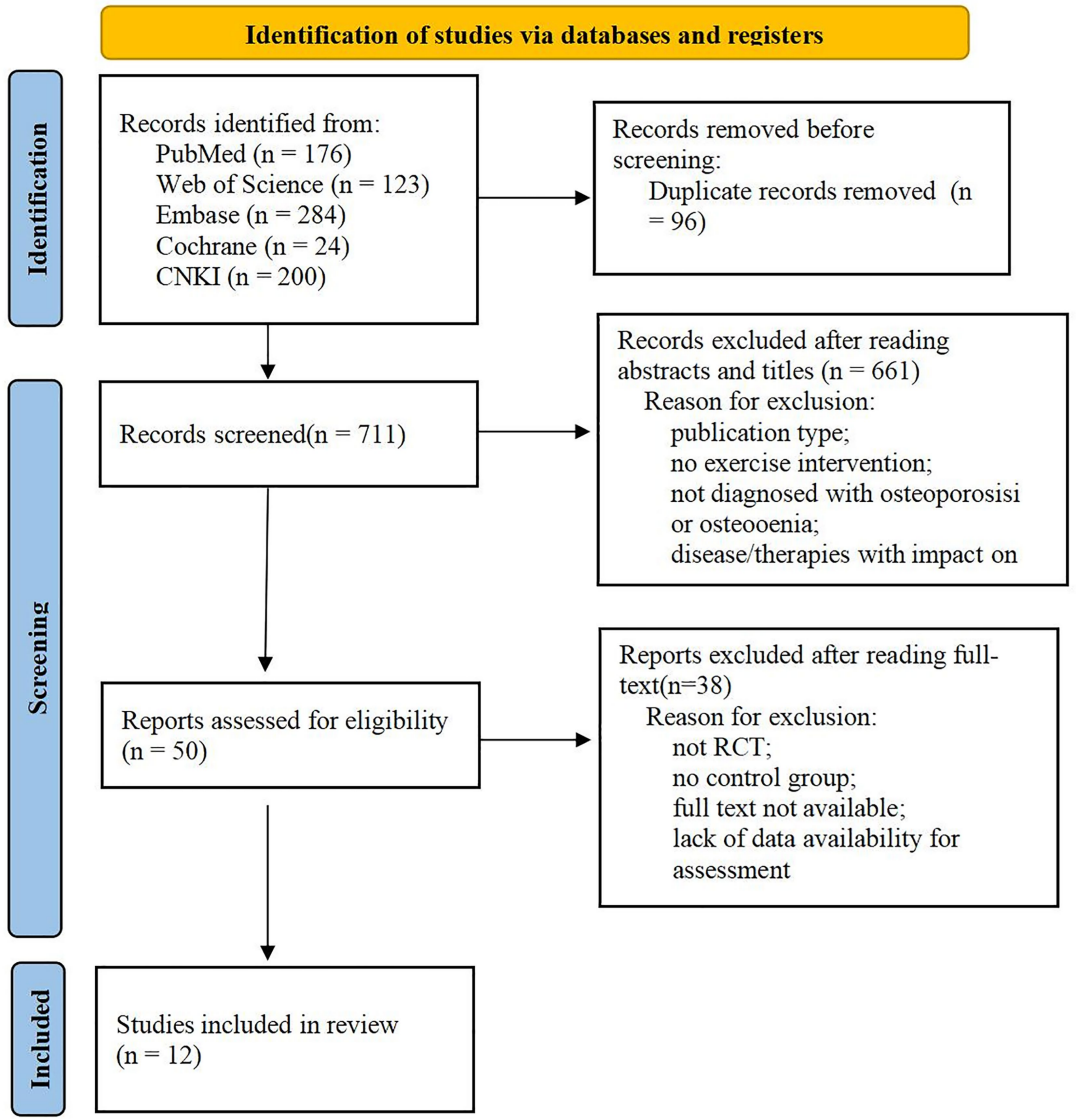
PRISMA flow diagram.

807 studies were initially identified from the selected databases; the document management software automatically deleted 96 duplicate entries. The remaining 711 studies were screened using the title and abstract, excluding another 661 studies. The remaining 50 studies were evaluated according to the inclusion and exclusion standards listed above. Ultimately, 12 RCTs were selected for our meta-analysis (18–29). See Figure 1 for the inclusion flow diagram. Two RCTs (19, 20) were defined as discrete research because of different follow-up times.

### Study characteristics and interventions

In 12 RCTs, the total number of participants was 1,289 (exercise group: *n* = 666; control group: *n* = 623) and sample sizes of the individual studies varied from 9 (25) to 216 (23). Of the 12 RCTs, eight (18–21, 24, 26, 28, 29) included only females (*n* = 724); and four (22, 23, 25, 27) included both sexes (M:F = 107:458). Age was closely associated with OVF (30). The mean age of participants in five RCTs (18–21, 23) was over 70 years, and it was lower than 70 years in seven (22, 24–29). All participants had been diagnosed with osteoporosis and had suffered at least one vertebral fracture, verified by MRI or CT. In 12 RCTs, the control groups were instructed to continue their current lifestyle. The intervention methods of the exercise groups were resistance and balance training. Four RCTs (22, 27–29) only evaluated resistance and balance training of the systematic lumbar and back muscles. Eight RCTs (18–21, 23–26) studied resistance and balance training of major muscle groups of the entire body and all four limbs. Three RCTs (18, 21, 26) involved aerobic training. The intervention duration varied from 4 weeks (28) to 1 year (24, 26). The frequency of prescribed training ranged from one session weekly (25) to two sessions daily (28). The prescribed training frequency was not specified in two studies (22, 27). Details of study characteristics and interventions are shown in Table 1.

**Table 1:** Characteristics of the included RCT studies.

First author, year	Study population	Sample size		Gender		Mean age		Exercise duration	Exercise intervention	Control intervention	Outcome
		Exercisers	Controls	Exercisers	Controls	Exercisers	Controls				
A Bergland, 2010	Wome*n* with osteoporosis and at least one vertebral fracture	47	42	F	F	70.8 ± 5.9	72 ± 5.8	2 sessions weekly, 3 months	Aerobic exercise to music (10 min) + Change direction to walk, climb, and avoid obstacles; balance training; chest and trunk exercise and posture promotion (40 min) + Stretching upper and lower limb muscles (10 min)	Maintain current lifestyle	A;B;C
B Stanghelle, 2020	Older women diagnosed with osteoporosis and vertebral fracture	76	73	F	F	74.7 ± 6.1	73.7 ± 5.6	2 sessions weekly, 3 months	Progressive resistance training for all major muscle groups combined with balance training.	Maintain current lifestyle	B;C;H;F
B Stanghelle, 2020	Older women diagnosed with osteoporosis and vertebral fracture	76	73	F	F	74.7 ± 6.1	73.7 ± 5.6	2 sessions weekly, 3 months	Group-based resistance and balance circuit program with instruction; focused on weight-bearing exercises.	Maintain current lifestyle	B;C;H;F
Chen, 2012	Older population with osteoporosis and at least one vertebral fracture	22	20	M:F 3:19	M:F 2:18	70.3 ± 14.1	67.1 ± 15.8	Unspecified	Systematic back muscle exercise with one-point, three-point, and five-point support training.	Maintain current lifestyle	E
C F Olsen, 2014	Older people with osteoporosis and at least one vertebral fracture	47	42	F	F	70.8 ± 5.9	72 ± 5.8	2 sessions weekly, 3 months	Aerobic exercise to music (10 min) + Change direction to walk, climb, and avoid obstacles; balance training; chest and trunk exercise and posture promotion (40 min) + Stretching upper and lower limb muscles (10 min)	Maintain current lifestyle	C; F
Ibolya Mikó, 2016	Older women with osteoporosis and at least one vertebral fracture	50	50	F	F	69.33 ± 4.6	69.1 ± 5.3	3 sessions weekly, 12 months	Combination program of conventional back, lower extremity and torso muscle strengthening and proprioceptive dynamic posture training; with three stages: static, dynamic, and functional phases.	Maintain current lifestyle	A
K L Barker, 2019	Older population with osteoporosis and at least one vertebral fracture	216	195	M:F 31:185	M:F 22:173	72.2 ± 8.4	71.9 ± 9.6	3–5 sessions weekly, 3 months	Pro program, multi-component of balance, strength training, and functional weight-bearing exercise	Single physiotherapy session	B; C; D;
Kim L Bennell, 2010	The older people with osteoporosis and at least one spinal fracture	11	9	M:F 4:7	M:F 0:9	66.2 ± 8.0	66.3 ± 11.8	1 session weekly, 10 weeks	Exercise for posture and range of motion, including standing, muscle contraction and extension, and resistance exercise	No additional intervention	A; B; D; E
L. Evstigneeva, 2016	Older women with osteoporosis and at least one vertebral fracture	40	38	F	F	70.7 ± 8.1	67.6 ± 7.0	2 sessions weekly, 12 months	Dynamic training for small and medium-sized muscle groups and limb joints, then dynamic exercise of equal length of major muscle groups and joints, then combined dynamic and breathing exercises	Maintain current level of physical activity	A; B
Wang, 2015	Older population with osteoporosis and at least one vertebral fracture	46	46	M:F 24:22	M:F 21:25	65.76 ± 5.3	66.74 ± 6.5	Unspecified	Progressive functional exercise of low back muscles: three-point and five-point support and flying swallow style training	Maintain current lifestyle	E
Yang, 2007	Older women with osteoporosis and at least one vertebral fracture	15	15	F	F	67.4 ± 5.6	65.6 ± 5.6	2 sessions daily, 4 weeks	Isometric contraction of lower back muscles in lying position; Bending and stretching training and rotation training of the waist in sitting or standing position	Maintain current lifestyle	A; C; E
Yetkin Çergel, 2019	Older women with osteoporosis and at least one vertebral fracture	20	20	F	F	58.90 ± 4.7	59.65 ± 6.5	3 sessions weekly, 6 weeks	Back extensor strengthening, with trunk extension, alternating arm lifts, opposite arm and leg lifts	Maintain daily activities	A; B; D; E

A: Time Up and Go (TUG), B: QUALEFFO-41, C: Functional reach, D: Kyphosis, E: visual analog scale (VAS), F: Falls efficacy scale international (FES-I).

### Methodology quality

**Figure 2:**
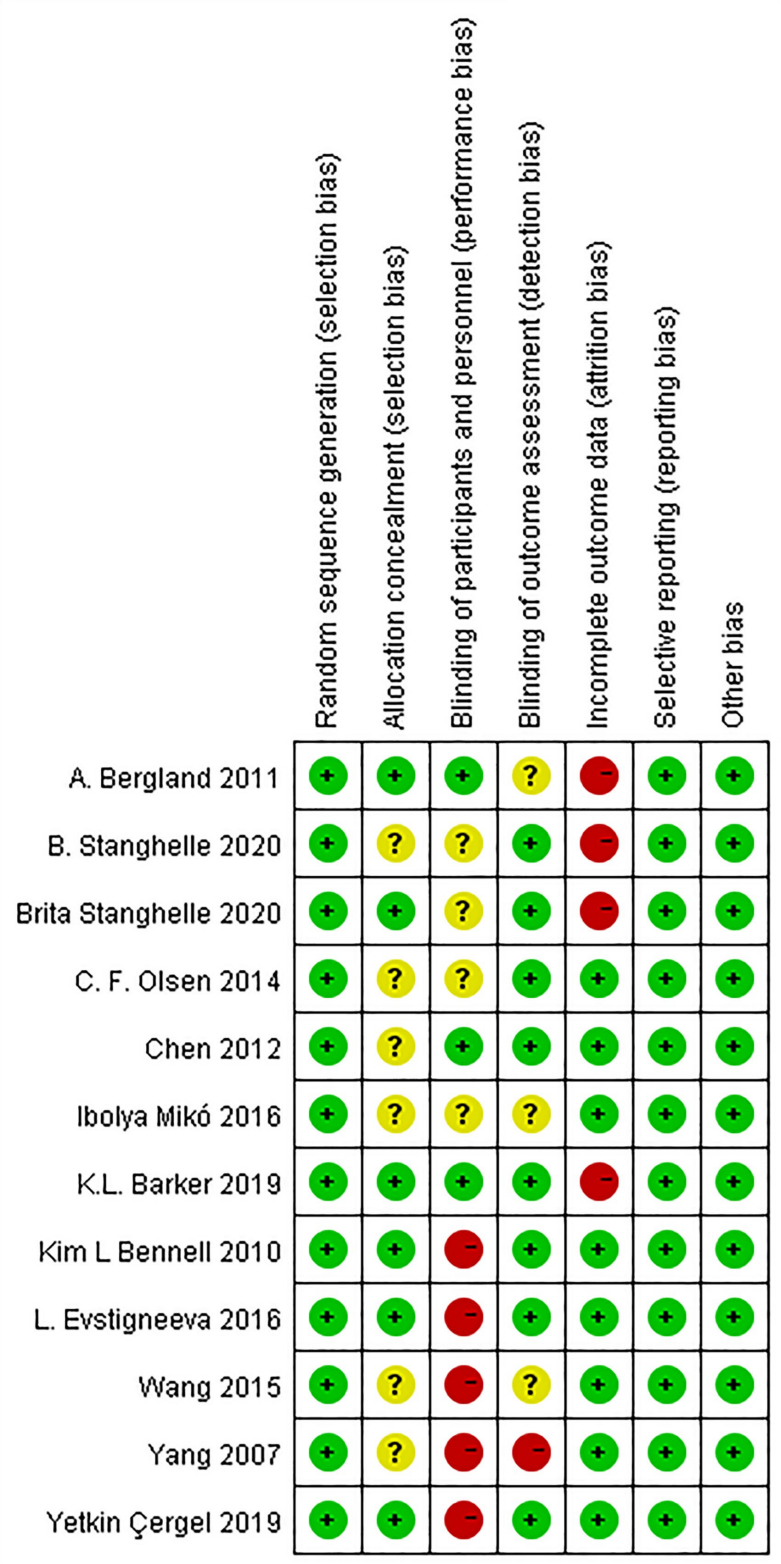
Risk of bias assessment summary of RCTs.

**Figure 3:**
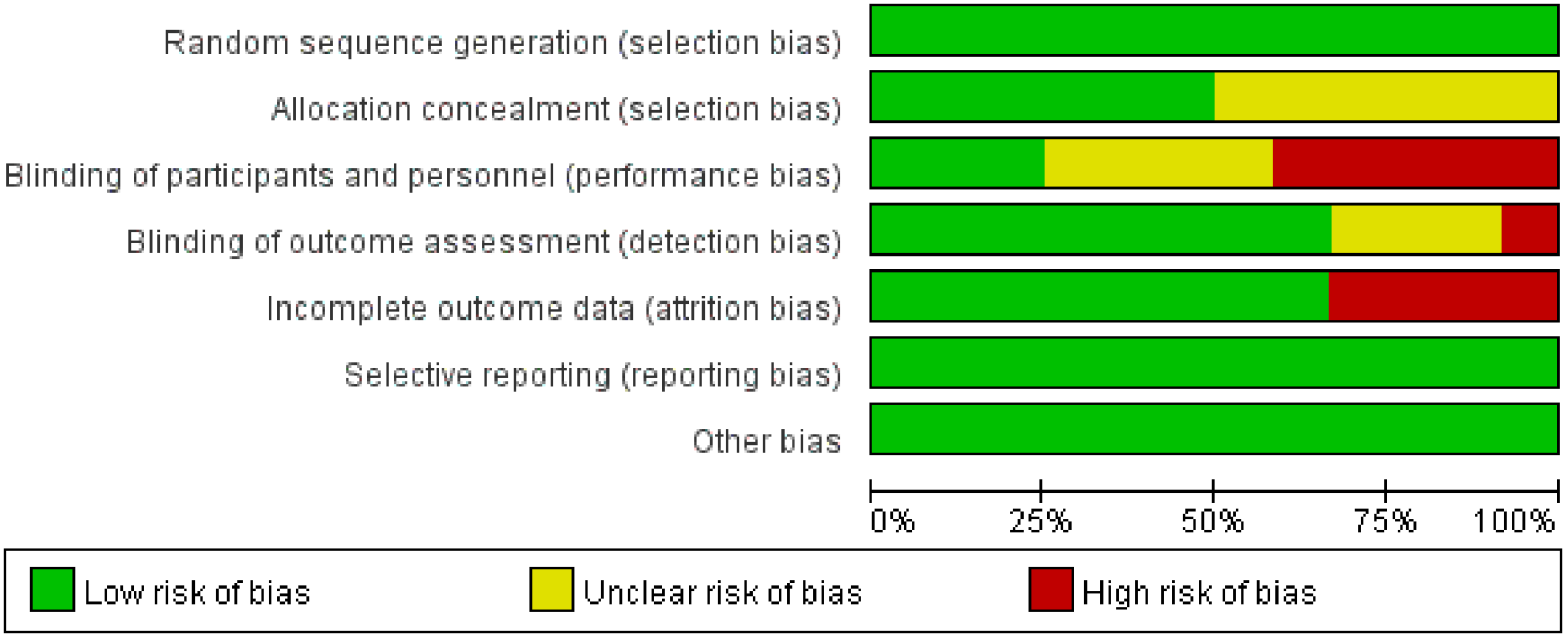
Risk of bias items as percentages across all included studies.

The Cochrane Collaboration tool was used to assess the RCT deviation risk on systematic review and meta-analysis. The results of the methodological quality assessment are shown in Figures 2, 3. All studies were judged as low risk of bias in the random sequence generation and selective reporting. Five studies were judged as high-risk of bias in the blinding of participants and personnel (25–29). All studies were judged at low risk of bias in the blinding of outcome assessments, except for one study (28) judged as high-risk. Four studies were judged as high-risk of bias for incomplete outcome data (18–20, 23). Risk of bias assessments are shown in Figures 2, 3.

## Outcome measures

### Effect of resistance and balance exercise training on QUALEFFO-41

**Figure 4:**
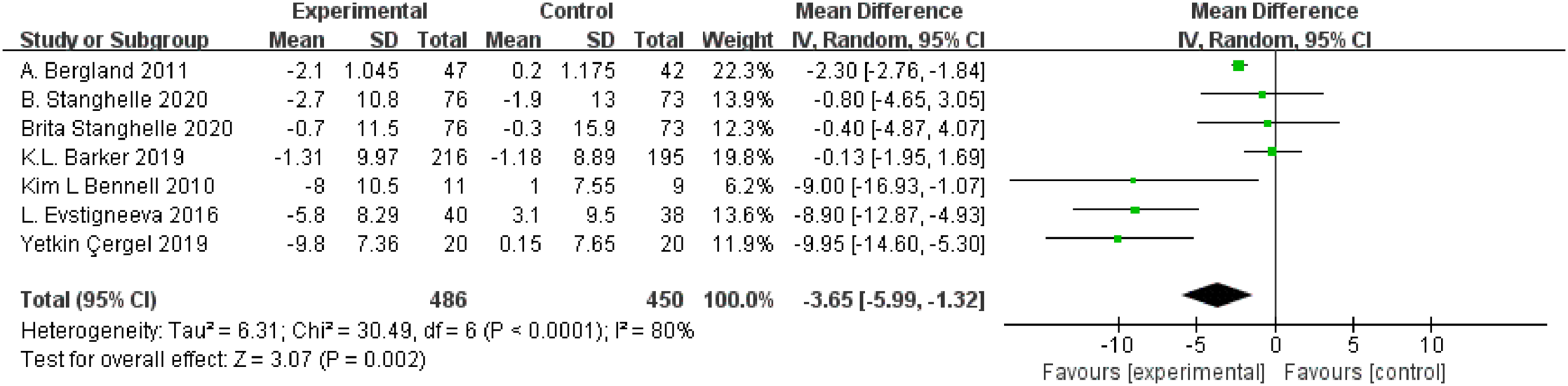
Forest plot for QUALEFFO-41 scores.

Seven RCTs (*n* = 936) assessed the effects of resistance and balance training on QUALEFFO-41 results (18–20, 23, 25, 26, 29). The seven RCT studies showed great heterogeneity (*I*^2^ = 80%, *p* < 0.0001, Chi^2^ = 6.31, df = 6); thus, the random effects model was used for analysis. Random effects analysis showed that resistance and balance training significantly decreased QUALEFFO-41 scores compared with those of the control group (mean deviation, MD: −3.65, 95% CI, −5.99 to −1.32, *p* = 0.002; Figure 4).

### Effect of resistance and balance exercise training On functional reach (FRT)

**Figure 5:**
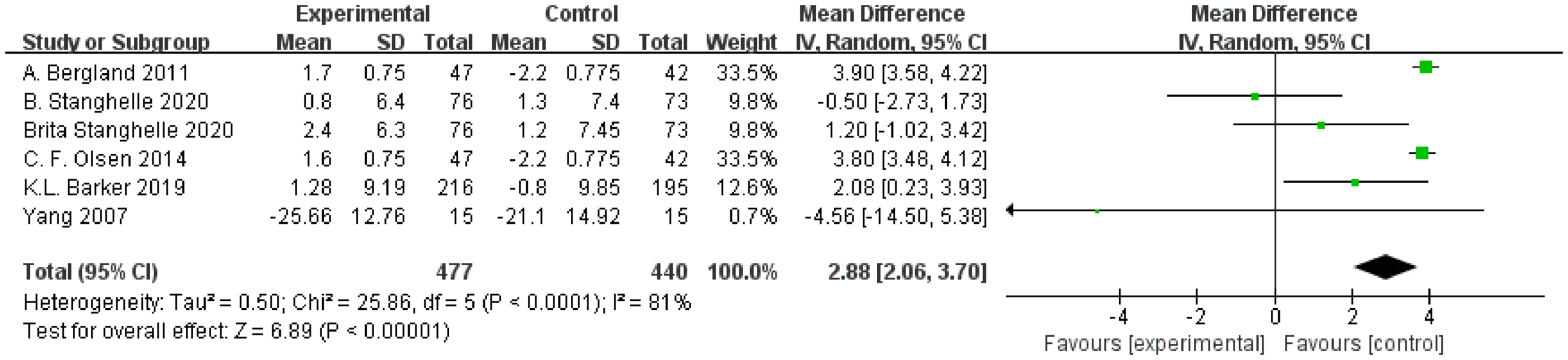
Forest plot for functional reach test (FRT).

The FRT was used to measure balancing ability. The FRT was used to measure the effects of resistance and balance training in six RCT studies (*n* = 917) (18–21, 23, 28). The heterogeneity among the studies was great (*I*^2^ = 81%, *p* < 0.0001, Chi^2^ = 25.86, df = 5); thus, the random effects model was used for analysis. Random effects analysis showed that the FRT was significantly increased in the group performing resistance and balance training versus the control group (MD: −1.59, 95% CI, −2.61 to −0.58, *p* = 0.002; Figure 5).

### Effect of resistance and balance exercise training on VAS

**Figure 6:**
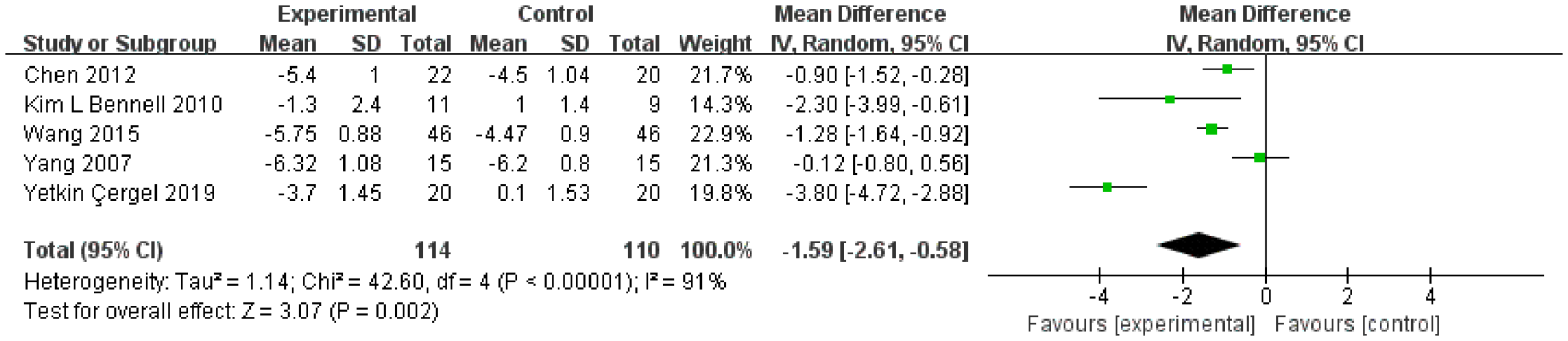
Forest plot for the VAS.

Lumbar back pain was a common complication of OVF. Five RCT studies used a VAS to assess the effect of resistance and balance training (*n* = 224) (22, 25, 27–29). The heterogeneity among the studies was great (*I*^2^ = 91%, *p* < 0.00001, Chi^2^ = 42.60, df = 4); therefore, we used the random effects model for analysis. Random effects analysis showed that the resistance and balance exercise significantly decreased VAS scores in the intervention group compared with controls (MD: −1.59, 95% CI, −2.61 to −0.58, *p* = 0.002; Figure 6).

### Effect of resistance and balance exercise training on TUG

**Figure 7:**
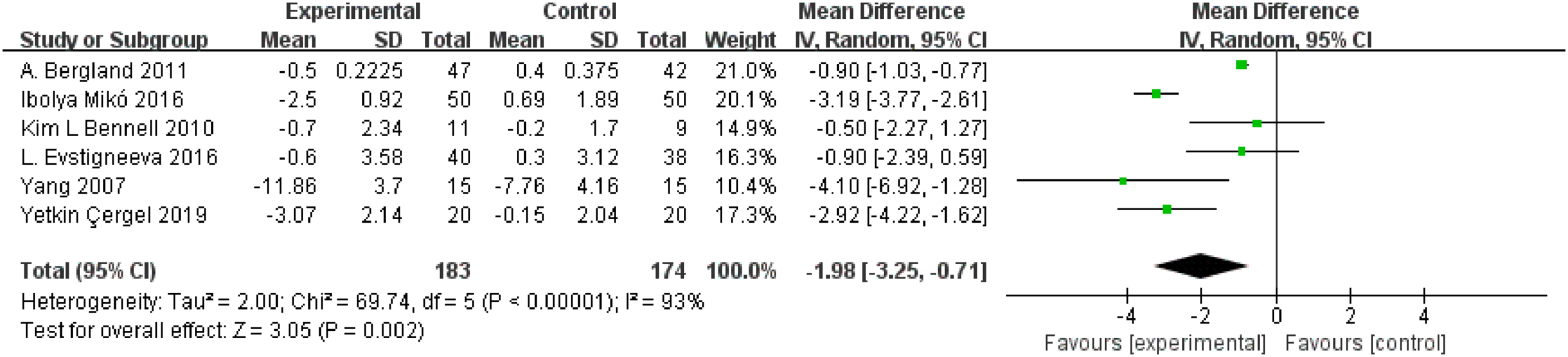
Forest plot for Timed Up and Go (TUG).

The TUG assessed functional mobility. Six RCTs (*n* = 357) assessed the effect of resistance and balance training on “Time Up and Go” (*n* = 357) (18, 24–26, 28, 29). There was great heterogeneity among the studies (*I*^2^ = 93%, *p* < 0.0001, Chi^2^ = 69.74, df = 5); therefore, the random effects model was used for analysis. Random effects analysis showed that TUG significantly decreased in the resistance and balance exercises group versus controls (MD: −1.98, 95% CI, −3.25 to −0.71, *p* = 0.002; Figure 7).

### Effect of resistance and balance exercise training on FES-I

**Figure 8:**
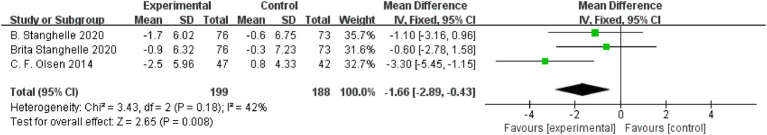
Forest plot for the falls efficacy scale international (FES-I).

FES-I was used to measure the degree of concern about falls during activities of daily living. Three RCT studies used the FES-I to assess the effect of resistance and balance training (*n* = 387) (19–21). The heterogeneity of the studies was normal (*I*^2^ = 42%); thus, a fixed effects model was used for analysis. Fixed effect analysis showed that FES-I was significantly decreased in the resistance and balance training group compared with the control group (MD: −1.66, 95% CI, −2.89 to −0.43, *p* = 0.008; Figure 8).

### Effect of resistance and balance exercise training on kyphosis

**Figure 9:**
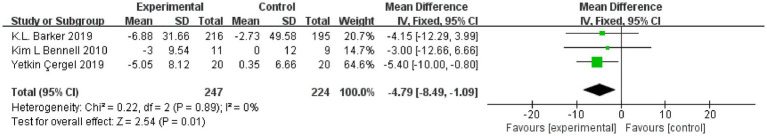
Forest plot for kyphosis.

Adverse consequences of kyphosis include injurious falls, fractures, functional limitations, mortality, and back pain. Three RCT studies used kyphosis to assess the effect of resistance and balance training (*n* = 471) (23, 25, 29). The heterogeneity among these studies was low (*I*^2^ = 0%); thus, the fixed effect model was used for analysis. Fixed effect analysis showed that kyphosis significantly decreased in the resistance and balance exercise group compared with the control group (MD: −4.79, 95% CI, −8.49 to −1.09, *p* = 0.01; Figure 9).

### Subgroup analysis

#### Age

We conducted subgroup analysis according to age (the mean age of the exercise and control groups combined). The included studies were divided into subgroups of under and over 70 years. Because all VAS studies included had means under 70 years, and those of FES-I were all over 70 years, the outcomes of these two were not included in the subgroup analysis. In the under 70 subgroup, comprehensive analysis showed that the exercise group was superior to the control group in QUALEFFO-41 (MD, −9.30, 95% CI, −12.12, −6.48); TUG (MD, −2.28, 95% CI, −3.48, 1.08); and kyphosis (MD, −4.96, 95% CI, −9.11, −0.80). There was no difference in functional reach (MD, −4.56, 95% CI, −14.50, 5.38). In the over 70 subgroup, the comprehensive analysis found that the training group was superior to the control group in QUALEFFO-41 (MD, −2.13 95% CI, −2.58, −1.69); TUG (MD, −0.90, 95% CI, −1.03, 0.77); and functional reach (MD, 2.97, 95% CI, 2.17, 3.70). There was no difference in kyphosis (MD, −4.15, 95% CI, −12.29, 3.99). Subgroup analysis results are shown in Table 2. The forest plots for subgroup analysis are shown in the Supplementary Documents.

**Table 2:** Subgroup analysis by age.

Outcome	Age	Included studies	Number	*I* ^2^	MD (95% CI)	*p*-value
QUALEFFO-41	<70	3	138	0%	−9.30 (−12.12, −6.48)	*p* < 0.00001
	>70	4	798	51%	−2.13 (−2.58, −1.69)	*p* < 0.00001
TUG	<70	4	268	74%	−2.28 (−3.48, 1.08)	*p* = 0.0002
	>70	2	89	0%	−0.90 (−1.03, 0.77)	*p* < 0.00001
Functional Reach	<70	1	30	0%	−4.56 (−14.50, 5.38)	*p* = 0.37
	>70	5	887	83%	2.97 (2.17, 3.70)	*p* < 0.00001
Kyphosis	<70	2	60	0%	−4.96 (−9.11, −0.80)	*p* = 0.02
	>70	1	411	0%	−4.15 (−12.29, 3.99)	*p* = 0.32

#### Exercise time

We conducted subgroup analysis of the primary outcomes according to exercise time. The included studies were divided into subgroups of under and over 10 weeks of exercise intervention. On the under 10 weeks subgroup analysis, the comprehensive results show that there was no significant difference between the exercise and control groups in the outcomes of functional reach (MD, −4.56, 95% CI, −14.50, 5.38) and VAS (MD, −1.95, 95% CI, −5.55, 1.66). However, the exercise group scored better than controls on the “QUALEFFO-41” (−2.67, 95% CI, −14.60, −5.30). On analysis of the over 10 weeks subgroup, the comprehensive results showed that the exercise group was superior to the control group in “QUALEFFO-41”(MD, −2.67, 95% CI, −4.82, −0.52); functional reach (MD, 2.97, 95% CI, 2.17, 3.76); and VAS (MD, −1.22, 95% CI,-1.64, −0.79). These subgroup analysis results are shown in Table 3. The forest plot for subgroup analysis are shown in the Supplementary material.

**Table 3:** Subgroup analysis by exercise time.

Outcome	Exercise time	Included studies	Number	*I* ^2^	MD (95% CI)	*p*-value
QUALEFFO-41	<10 weeks	1	40	0%	−2.67 (−14.60, −5.30)	*p* < 0.0001
	>10 weeks	6	896	75%	−2.67 (−4.82, −0.52)	*p* = 0.02
Functional Reach	<10 weeks	1	30	0%	−4.56 (−14.50, 5.38)	*p* = 0.37
	>10 weeks	5	887	83%	2.97 (2.17, 3.76)	*p* < 0.00001
VAS	<10 weeks	2	70	97%	−1.95 (−5.55, 1.66)	*p* = 0.29
	>10 weeks	3	154	26%	−1.22 (−1.64, −0.79)	*p* < 0.00001

#### Publication bias

We planned to use a funnel chart to evaluate publication bias, but the number of included trials was few (*n* = 12), and the number of patients per trial was also too few (9–216). Thus, we were unable to effectively evaluate publication bias.

## Discussion

The main purpose of this study was to evaluate the effects of resistance and balance exercise training on functional status of patients with OVF, through a systematic review and meta-analysis of RCTs. We identified 12 RCTs of patients with OVF using resistance and balance training as the intervention, with functional status and healthy quality of life as the outcomes. For patients with OVF, resistance and balance training ameliorated functional activity, improved body balance, and reduced the degree of back pain. In addition, the positive effect on OVF was seen only when the resistance and balance training lasted for over 10 weeks, and it has little relationship with the patient’s age.

The outcome measurements identified in this review were in two primary areas: physical activity and balance capacity. In this meta-analysis, the primary outcomes were scores of QUALEFFO-41, VAS, and functional reach. QUALEFFO-41 assesses quality of life in terms of physical function (17 items), pain (5 items), social function (7 items), mental health (9 items) and general health (3 items) (31). At present, it has become one of the most important indicators to evaluate quality of life in patients with osteoporosis (32). In this meta-analysis, resistance and balance exercise comparatively reduced the QUALEFFO-41 (MD:-3.65, 95% CI, −5.99 to −1.32, *p* = 0.002) in the exercise group, clearly demonstrating the positive significance of the exercise program on quality of life, physical function, and other aspects of OVF patients’ lives. On subgroup analysis, the final outcomes had no significant relationship with age or exercise time. Therefore, patients with OVF should actively perform resistance and balance training, and they should perform it for longer than 10 weeks. Evstigneeva et al. (26) found that resistance and balance training not only significantly reduced the total scores of QUALEFFO-41 but also had an optimistic impact on the individual score of QUALEFFO-41 (26). Functional Reach (FR) is a clinical measurement method to evaluate dynamic balance (33). In this meta-analysis, resistance and balance exercise increased the FR (a better result) (MD:-1.59, 95% CI, −2.61 to −0.58, *p* = 0.002) of the exercise group, clearly demonstrating the dynamic balance ability that exercise programs can create for patients with OVF. The under 70 years and the under 10 weeks subgroups showed no significant differences on subgroup analysis. On subgroup analysis of the over 70 years and over 10 weeks, the exercise group showed superior FR (age MD, 2.97, 95% CI, 2.17, 3.70; exercise time, MD, 2.97, 95% CI, 2.17, 3.70) to that of the control group. Two subgroups were included within the same outcomes. Previously, Watson et al. studied the impact of 8 months of resistance and impact exercise on postmenopausal women with osteoporosis. Compared with scores of the control group (5.4 ± 7.2% versus 0.1 ± 7.2%, *p* < 0.001), the FR was significantly increased in the exercise group (95% CI 3.4% to 7.5% versus–1.8 to 2.1%) (34).This is consistent with our results. Pain was one of the most common clinical symptoms in patients with OVF (35). On this meta-analysis, VAS pain scores (MD, −1.59, 95%CI, −2.61 to −0.58, *p* = 0.002) decreased significantly after resistance and balance training, and the exercise group was significantly better than the controls. On subgroup analysis of under 10 weeks, VAS did not differ in the exercise and control groups. In a subgroup analysis of more than 10 weeks, VAS scores were superior in the exercise group (Exercise time MD, −1.22, 95% CI, −1.64, −0.79) versus the control group. Lyles et al. (35) found that pain led to significantly slower walking speed and greater postural swing. Furthermore, pain reduced muscle strength and increased patients’ fear of falling. Therefore, among patients with OVF, pain both increased the fear of falling and decreased physical performance (36). In this study, the secondary outcomes were TUG, kyphosis and FES-I. TUG is a dynamic balance assessment tool that assesses the quality and strength of muscles and has been used to predict repeated falls (37). Increased kyphosis angle is considered to represent the presence of osteoporosis; it also damages balance and postural stability, reduces gait stability, and increases the risk of falls (38). FES-I has been used to assess the fear of falling in daily sports and social activities, and was closely related to physical balance (39). Therefore, secondary outcomes were used to predict fall risk and were closely related to body balance. In this meta-analysis, the secondary outcomes were significantly better in the exercise versus control group (walking speed MD, −1.26, 95% CI, −1.83 to −0.68, *p* < 0.0001; TUG MD, −1.98, 95% CI, −3.25 to −0.71, *p* = 0.002; FES-I MD, −1.66, 95% CI, −2.89 to −0.43, *p* = 0.008; kyphosis MD, −4.79, 95%CI, −8.49 to −1.09, *p* = 0.01). OVF is often accompanied by back pain, hunchback, motor dysfunction, and psychological distress, including anxiety, depression, and fear. Through the above indicators, we found that patients with OVF who performed resistance and balance training experienced significantly reduced pain and fear of falling, improved motor and balance function, and ultimately, may reduce repeat falls risk. The intervention had positive significance for patients’ physiological and psychological improvement overall.

Few studies are currently assessing the effects of exercise programs on patients with OVF. Cochrane recently published a review in this area but did not reach a definitive conclusion (40). In the review, Gibbs et al. (40) studied nine randomized controlled trials or semi-randomized trials to evaluate the benefits and hazards of exercise intervention for 4 weeks or more on patients with OVF. They assessed the incidence of re-fracture, pain, falls, health-related quality of life, physical function, and adverse events. They concluded that exercise might improve the patient’s physical fitness, but they did not determine the impact of exercise on falls, accidental fractures, or adverse events. This contrasted with our results. One explanation for the divergence in our results and the data of Gibbs et al. (40) was that the exercise program they investigated was non-specific. The type and intensity of exercise cannot be ignored (40–42). The review by Gibbs et al. (40) involved any type of exercise, including resistance training, balance training, aerobic exercise, tai chi, or other personalized programs. This non-specificity would mean greater results heterogeneity and thus, influence the conclusion. Although there have been some evidence-based recommendations (43, 44) for exercise programs, an optimal program has not yet been determined. The latest clinical prevention and treatment guidelines for osteoporosis strongly recommend that patients carry out a multicomponent program including resistance and balance training and back muscle exercise plans (45). Such an exercise program should enhance muscle strength, improve balance, improve functional status, and ultimately reduce the risk of falls (46, 47). Falling is well known as an important risk factor for fracture (48). For female patients with osteoporosis, falls will increase the risk of spinal fracture 2.5-fold and hip fracture 3.1-fold (49). Therefore, patients with OVF need to perform more resistance and balance exercise; this is consistent with our recommendations.

A resistance and balance training program can improve muscle strength, quality, functional activity, and cardiopulmonary function (50–52) and reduce the risk of falls in older populations (53). Resistance and balance training programs have also positively impacted many diseases, including stroke (54), Parkinson’s disease (55), hypertension (56), musculoskeletal pain (57), cardiovascular disease (58), and anxiety symptoms (59). In our review, the intervention program was limited to resistance and balance exercise programs, which reduced the degree of low back pain and improved physical function and quality of life. However, on completing our research, it was clear that there was limited information available regarding resistance and balance motion programs and patients with OVF. To date, there is no specific exercise scheme. Given the complexity of these issues, research conducted within this area appears to lack consistency. Therefore, we explored one specific exercise scheme. In this program, patients were required to complete some warm-up aerobic exercise before the official start. In this movement stage, small and medium-sized muscle groups, and limb joints moved dynamically. The patients began formal exercise after 10 min of warm-up. For this, stood on different surfaces with one leg; the other leg performed various movements to improve their physical balance. The patients could perform chest presses and biceps curls with suitable dumbbells or carry out upright rows for upper back strength and posture. During these activities, the patient actively contracts the scapula and back muscles. This was followed by a series of exercises for enhancing lower extremity muscle strength, e.g., walking backwards, forwards and sideways, climbing steps and performing squats (holding dumbbells or not). Back muscle training developed gradually into five-point, three-point, and one-point support training, progressing from simple to difficult. In this stage, the main muscle groups and joints performed dynamic movements, which enhanced the strength and function of the muscles of the extremities, abdomen, and waist, and improved posture. The program ended with a cool-down period, stretching the muscles of the limbs. In addition to the resistance and balance training, we recommended that patients should exercise for 10 weeks, as soon as possible under the supervision and guidance of medical professionals; this might reduce the probability of falls.

There were limitations to our meta-analysis. The quality of research methods included in this review was variable, but overall, quality was medium to high. Nonetheless, since all studies were observational, there was still the possibility of bias and/or confusion. There were too few studies (*n* = 12) to adequately assess publication bias. On the other hand, due to the small number of studies included, we were only able to conduct subgroup analysis on the primary outcomes. Fortunately, the subgroup analysis supported our conclusions. In addition, the starting time for the resistance and balance training the severity of vertebral fracture (number of vertebral fractures and reduction in body weight) in OVF patients were not clear; this contributed substantially to heterogeneity. In the future, we will seek to identify the correlation of treatment effects with the start time of exercise intervention. It would also be of interest to evaluate outcome measures besides symptom rating scales, such as bone mineral density, cost-effectiveness, or to show heterogeneity of treatment effects.

## Conclusion

The resistance and balance exercise training enhanced muscle strength, improved functional activity and balance, reduced pain and fear of falling, which may prevents falls in patients with OVF. For patients of any age with OVF, a resistance and balance exercise training program lasting at least 10 weeks and beginning as early as possible will be beneficial regarding quality of life and activities of daily living. For future research, we will investigate a specific exercise scheme. We will aim to determine the best time for patients to begin the resistance and balance training, and we plan a multicenter, large sample RCT to determine the positive effects of this program.

## Data availability statement

The raw data supporting the conclusions of this article will be made available by the authors, without undue reservation.

## Author contributions

XL and ZH conceived the idea for this paper, wrote the protocol, conducted the literature review, and contributed to writing. WC, QC, and FFL extracted the data and edited the manuscript. CC, PL, and FYL conducted research quality assessment. SG and PC conducted a statistical analysis. WY, DL, and SW contributed to writing and editing. All authors contributed to the article and approved the submitted version.

## Funding

This study was funded by the National Key Research and Development Program (no: 2019YFC1709905), Three year Action Plan of Shanghai to Further Accelerate the Inheritance, Innovation and Development of Traditional Chinese Medicine (ZY(2021-2023)-0201-01), Pudong New Area Health System Pudong Famous Physician Training Plan (PWRzm2020-15) and Xuhui District Artificial Intelligence Medical Hospital Local Cooperation Project (2021-016).

## Conflict of interest

The authors declare that the research was conducted in the absence of any commercial or financial relationships that could be construed as a potential conflict of interest.

## Publisher’s note

All claims expressed in this article are solely those of the authors and do not necessarily represent those of their affiliated organizations, or those of the publisher, the editors and the reviewers. Any product that may be evaluated in this article, or claim that may be made by its manufacturer, is not guaranteed or endorsed by the publisher.

## References

[1] Reid IR Billington EO. Drug therapy for osteoporosis in older adults. Lancet. (2022) 399:1080–92. doi: 10.1016/S0140-6736(21)02646-5 35279261

[2] Cummings SR Melton LJ. Epidemiology and outcomes of osteoporotic fractures. Lancet. (2002) 359:1761–7. doi: 10.1016/S0140-6736(02)08657-9 12049882

[3] Wang LH Yu W Yin XJ Cui LJ Tang SY Jiang N. Prevalence of osteoporosis and fracture in China: the China osteoporosis prevalence study. JAMA Netw Open. (2021) 4:e2121106. doi: 10.1001/jamanetworkopen.2021.21106, PMID: 34398202 PMC8369359

[4] Capdevila-Reniu A Navarro-López M López-Soto A. Osteoporotic vertebral fractures: a diagnostic challenge in the 21ST century. Rev Clin Esp. (2019) S0014-2565:30240–1. doi: 10.1016/j.rceng.2019.09.013 33998487

[5] Singer A Exuzides A Spangler L O'Malley C Colby C Johnston K. Burden of illness for osteoporotic fractures compared with other serious diseases among postmenopausal women in the United States. Mayo Clin Proc. (2015) 90:53–62. doi: 10.1016/j.mayocp.2014.09.011, PMID: 25481833

[6] Kendler DL Bauer DC Davison KS Dian L Hanley DA Harris ST. Vertebral fractures: clinical importance and management. Am J Med. (2016) 129:e1–e10. doi: 10.1016/j.amjmed.2015.09.020 26524708

[7] Johansson H Siggeirsdóttir K Harvey NC Odén A Gudnason V McCloskey E. Imminent risk of fracture after fracture. Osteoporos Int. (2017) 28:775–80. doi: 10.1007/s00198-016-3868-0, PMID: 28028554 PMC5338733

[8] Johansson H Odén A McCloskey EV Kanis JA. Mild morphometric vertebral fractures predict vertebral fractures but not non-vertebral fractures. Osteoporos Int. (2014) 25:235–41. doi: 10.1007/s00198-013-2460-0 23974856

[9] Yu WY Hwang HF Chen CY Lin MR. Situational risk factors for fall-related vertebral fractures in older men and women. Osteoporos Int. (2021) 32:1061–70. doi: 10.1007/s00198-020-05799-x, PMID: 33415375

[10] Singh MA. Exercise comes of age: rationale and recommendations for a geriatric exercise prescription. J Gerontol A Biol Sci Med Sci. (2002) 57:M262–82. doi: 10.1093/gerona/57.5.M262, PMID: 11983720

[11] Beck BR Daly RM Singh MAF Taaffe DR. Exercise and sports science Australia (ESSA) position statement on exercise prescription for the prevention and management of osteoporosis. J Sci Med Sport. (2017) 20:438–45. doi: 10.1016/j.jsams.2016.10.001, PMID: 27840033

[12] Sanchez-Trigo H Rittweger J Sañudo B. Effects of non-supervised exercise interventions on bone mineral density in adult women: a systematic review and meta-analysis. Osteoporos Int. (2022) 33:1415–27. doi: 10.1007/s00198-022-06357-3, PMID: 35218402 PMC8881760

[13] Varahra A Rodrigues IB MacDermid JC Bryant D Birmingham T. Exercise to improve functional outcomes in persons with osteoporosis: a systematic review and meta-analysis. Osteoporos Int. (2018) 29:265–86. doi: 10.1007/s00198-017-4339-y, PMID: 29306984

[14] Giangregorio LM Papaioannou A MacIntyre NJ Ashe MC Heinonen A Shipp K. Too fit to fracture: exercise recommendations for individuals with osteoporosis or osteoporotic vertebral fracture. Osteoporos Int. (2014) 25:821–35. doi: 10.1007/s00198-013-2523-2, PMID: 24281053 PMC5112023

[15] Sinaki M Itoi E Wahner HW Wollan P Gelzcer R Mullan BP. Stronger back muscles reduce the incidence of vertebral fractures: a prospective 10 year follow-up of postmenopausal women. Bone. (2002) 30:836–41. doi: 10.1016/S8756-3282(02)00739-1, PMID: 12052450

[16] Giangregorio LM Macintyre NJ Thabane L Skidmore CJ Papaioannou A. Exercise for improving outcomes after osteoporotic vertebral fracture. Cochrane Database Syst Rev. (2013) 1:CD008618. doi: 10.1002/14651858.CD008618.pub2 PMC5104540 23440829

[17] Page MJ McKenzie JE Bossuyt PM Boutron I Hoffmann TC Mulrow CD. The PRISMA 2020 statement: an updated guideline for reporting systematic reviews. Syst Rev. (2021) 10:89–100. doi: 10.1186/s13643-021-01626-4, PMID: 33781348 PMC8008539

[18] Bergland A Thorsen H Kåresen R. Effect of exercise on mobility, balance, and health-related quality of life in osteoporotic women with a history of vertebral fracture: a randomized, controlled trial. Osteoporos Int. (2011) 22:1863–71. doi: 10.1007/s00198-010-1435-7, PMID: 21060992

[19] Stanghelle B Bentzen H Giangregorio L Pripp AH Skelton DA Bergland A. Effects of a resistance and balance exercise programme on physical fitness, health-related quality of life and fear of falling in older women with osteoporosis and vertebral fracture: a randomized controlled trial. Osteoporos Int. (2020) 31:1069–78. doi: 10.1007/s00198-019-05256-4, PMID: 31925473

[20] Stanghelle B Bentzen H Giangregorio L Pripp AH Skelton DA Bergland A. Physical fitness in older women with osteoporosis and vertebral fracture after a resistance and balance exercise programme: 3-month post-intervention follow-up of a randomised controlled trial. BMC Musculoskelet Disord. (2020) 21:471–82. doi: 10.1186/s12891-020-03495-9, PMID: 32682416 PMC7368978

[21] Olsen CF Bergland A. The effect of exercise and education on fear of falling in elderly women with osteoporosis and a history of vertebral fracture: results of a randomized controlled trial. Osteoporos Int. (2014) 25:2017–25. doi: 10.1007/s00198-014-2724-3, PMID: 24807628

[22] Chen BL Zhong Y Huang YL Zeng LW Li YQ Yang XX. Systematic back muscle exercise after percutaneous vertebroplasty for spinal osteoporotic compression fracture patients: a randomized controlled trial. Clin Rehabil. (2012) 26:483–92. doi: 10.1177/0269215511423557, PMID: 21975470

[23] Barker KL Newman M Stallard N Leal J Lowe CM Javaid MK. Physiotherapy rehabilitation for osteoporotic vertebral fracture-a randomised controlled trial and economic evaluation (PROVE trial). Osteoporos Int. (2020) 31:277–89. doi: 10.1007/s00198-019-05133-0, PMID: 31720722

[24] Mikó I Szerb I Szerb A Poor G. Effectiveness of balance training programme in reducing the frequency of falling in established osteoporotic women: a randomized controlled trial. Clin Rehabil. (2017) 31:217–24. doi: 10.1177/0269215516628616, PMID: 26825109

[25] Bennell KL Matthews B Greig A Briggs A Kelly A Sherburn M. Effects of an exercise and manual therapy program on physical impairments, function and quality-of-life in people with osteoporotic vertebral fracture: a randomised, single-blind controlled pilot trial. BMC Musculoskelet Disord. (2010) 11:36–47. doi: 10.1186/1471-2474-11-36, PMID: 20163739 PMC2830179

[26] Evstigneeva L Lesnyak O Bultink IE Lems WF Kozhemyakina E Negodaeva E. Effect of twelve-month physical exercise program on patients with osteoporotic vertebral fractures: a randomized, controlled trial. Osteoporos Int. (2016) 27:2515–24. doi: 10.1007/s00198-016-3560-4, PMID: 26984569

[27] Wang XF Xu B Ye XY Yang YH Wang GH. Effects of different treatments on patients with osteoporotic fracture after percutaneous kyphoplasty. Zhong Guo Gu Shang. (2015) 28:512–6. 26255474

[28] Yang L He CQ Lei ZJ Xie W Lan Q. Effect of pain-free exercises on female osteoporosis patients with spinal compressive fracture. CJTER. (2017) 11:9108–11.

[29] Çergel Y Topuz O Alkan H Sarsan A Sabir AN. The effects of short-term back extensor strength training in postmenopausal osteoporotic women with vertebral fractures: comparison of supervised and home exercise program. Arch Osteoporos. (2019) 14:82–90. doi: 10.1007/s11657-019-0632-z, PMID: 31352573

[30] Rubin KH Rothmann MJ Holmberg T Høiberg M Möller S Barkmann R. Effectiveness of a two-step population-based osteoporosis screening program using FRAX: the randomized risk-stratified osteoporosis strategy evaluation (ROSE) study. Osteoporos Int. (2018) 29:567–78. doi: 10.1007/s00198-017-4326-3, PMID: 29218381

[31] Lips P Cooper C Agnusdei D Caulin F Egger P Johnell O. Quality of life as outcome in the treatment of osteoporosis: the development of a questionnaire for quality of life by the European Foundation for Osteoporosis. Osteoporos Int. (1997) 7:36–8. doi: 10.1007/BF01623457, PMID: 9102060

[32] Nuti R Caffarelli C Guglielmi G Gennari L Gonnelli S. Undiagnosed vertebral fractures influence quality of life in postmenopausal women with reduced ultrasound parameters. Clin Orthop Relat Res. (2014) 472:2254–61. doi: 10.1007/s11999-014-3588-8, PMID: 24728662 PMC4048413

[33] Wernick-Robinson M Krebs DE Giorgetti MM. Functional reach: does it really measure dynamic balance? Arch Phys Med Rehabil. (1999) 80:262–9. doi: 10.1016/S0003-9993(99)90136-3 10084433

[34] Watson S Weeks B Weis L Harding A Horan S Beck B. High-intensity resistance and impact training improves bone mineral density and physical function in postmenopausal women with osteopenia and osteoporosis: the LIFTMOR randomized controlled trial. J Bone Miner Res. (2019) 34:572. doi: 10.1002/jbmr.3659, PMID: 30861219

[35] Lyles KW Gold DT Shipp KM Pieper CF Martinez S Mulhausen PL. Association of osteoporotic vertebral compression fractures with impaired functional status. Am J Med. (1993) 94:595–601. doi: 10.1016/0002-9343(93)90210-G, PMID: 8506884

[36] Hübscher M Vogt L Schmidt K Fink M Banzer W. Perceived pain, fear of falling and physical function in women with osteoporosis. Gait Posture. (2010) 32:383–5. doi: 10.1016/j.gaitpost.2010.06.018, PMID: 20663672

[37] YuXHouLGuoJWangYHanPFuL. Combined effect of osteoporosis and poor dynamic balance on the incidence of sarcopenia in elderly Chinese community suburban-dwelling individuals. J Nutr Health Aging. (2020) 24:71–7. doi: 10.1007/s12603-019-1295-6, PMID: 31886811

[38] Koelé MC Lems WF Willems HC. The clinical relevance of Hyperkyphosis: a narrative review. Front Endocrinol (Lausanne). (2020) 11:5–12. doi: 10.3389/fendo.2020.00005, PMID: 32038498 PMC6993454

[39] Halvarsson A Franzén E Ståhle A. Assessing the relative and absolute reliability of the falls efficacy scale-international questionnaire in elderly individuals with increased fall risk and the questionnaire's convergent validity in elderly women with osteoporosis. Osteoporos Int. (2013) 24:1853–8. doi: 10.1007/s00198-012-2197-1, PMID: 23124715

[40] Gibbs JC MacIntyre NJ Ponzano M Templeton JA Thabane L Papaioannou A. Exercise for improving outcomes after osteoporotic vertebral fracture. Cochrane Database Syst Rev. (2019) 7:CD008618. doi: 10.1002/14651858.CD008618.pub3, PMID: 31273764 PMC6609547

[41] Kemmler W Shojaa M Kohl M von Stengel S. Effects of different types of exercise on bone mineral density in postmenopausal women: a systematic review and meta-analysis. Calcif Tissue Int. (2020) 107:409–39. doi: 10.1007/s00223-020-00744-w, PMID: 32785775 PMC7546993

[42] Kistler-Fischbacher M Weeks BK Beck BR. The effect of exercise intensity on bone in postmenopausal women (part 2): a meta-analysis. Bone. (2021) 143:115697–719. doi: 10.1016/j.bone.2020.115697, PMID: 33357834

[43] Brooke-Wavell K Skelton DA Barker KL Clark EM De BS Arnold S. Strong, steady and straight: UK consensus statement on physical activity and exercise for osteoporosis. Br J Sports Med. (2022) 56:837–46. doi: 10.1136/bjsports-2021-104634, PMID: 35577538 PMC9304091

[44] Harding AT Weeks BK Lambert C Watson SL Weis LJ Beck BR. Exploring thoracic kyphosis and incident fracture from vertebral morphology with high-intensity exercise in middle-aged and older men with osteopenia and osteoporosis: a secondary analysis of the LIFTMOR-M trial. Osteoporos Int. (2021) 32:451–65. doi: 10.1007/s00198-020-05583-x, PMID: 32935171

[45] LeBoff MS Greenspan SL Insogna KL Lewiecki EM Saag KG Singer AJ. The clinician's guide to prevention and treatment of osteoporosis. Osteoporos Int. (2022) 33:2049–102. doi: 10.1007/s00198-021-05900-y, PMID: 35478046 PMC9546973

[46] Senderovich H Tang H Belmont S. The role of exercises in osteoporotic fracture prevention and current care gaps. Where are we now? Recent updates. Rambam Maimonides Med J. (2017) 8:e0032–46. doi: 10.5041/RMMJ.10308, PMID: 28786812 PMC5548111

[47] Sherrington C Fairhall N Wallbank G Tiedemann A Michaleff ZA Howard K. Exercise for preventing falls in older people living in the community: an abridged Cochrane systematic review. Br J Sports Med. (2020) 54:885–91. doi: 10.1136/bjsports-2019-101512, PMID: 31792067

[48] Barron RL Oster G Grauer A Crittenden DB Weycker D. Determinants of imminent fracture risk in postmenopausal women with osteoporosis. Osteoporos Int. (2020) 31:2103–11. doi: 10.1007/s00198-020-05294-3, PMID: 32613410 PMC7560920

[49] Kim KM Lui LY Cummings SR. Recent fall and high imminent risk of fracture in older men and women. Age Ageing. (2022) 51:afac141. doi: 10.1093/ageing/afac141, PMID: 35753766 PMC9233980

[50] Fyfe JJ Hamilton DL Daly RM. Minimal-dose resistance training for improving muscle mass, strength, and function: a narrative review of current evidence and practical considerations. Sports Med. (2022) 52:463–79. doi: 10.1007/s40279-021-01605-8, PMID: 34822137

[51] Aartolahti E Lönnroos E Hartikainen S Häkkinen A. Long-term strength and balance training in prevention of decline in muscle strength and mobility in older adults. Aging Clin Exp Res. (2020) 32:59–66. doi: 10.1007/s40520-019-01155-0, PMID: 30830597 PMC6974487

[52] Tamulevičiūtė-Prascienė E Beigienė A Thompson MJ Balnė K Kubilius R Bjarnason-Wehrens B. The impact of additional resistance and balance training in exercise-based cardiac rehabilitation in older patients after valve surgery or intervention: randomized control trial. BMC Geriatr. (2021) 21:23–35. doi: 10.1186/s12877-020-01964-3, PMID: 33413144 PMC7792183

[53] Mañas A Gómez-Redondo P Valenzuela PL Morales JS Lucía A Ara I. Unsupervised home-based resistance training for community-dwelling older adults: a systematic review and meta-analysis of randomized controlled trials. Ageing Res Rev. (2021) 69:101368–72. doi: 10.1016/j.arr.2021.101368, PMID: 34022464

[54] Saunders DH Sanderson M Hayes S Johnson L Kramer S Carter DD. Physical fitness training for stroke patients. Cochrane Database Syst Rev. (2020) 2020:CD003316–628. doi: 10.1002/14651858.CD003316.pub7 PMC7083515 32196635

[55] Gollan R Ernst M Lieker E Caro-Valenzuela J Monsef I Dresen A. Effects of resistance training on motor-and non-motor symptoms in patients with Parkinson's disease: a systematic review and meta-analysis. J Parkinsons Dis. (2022) 12:1783–806. doi: 10.3233/JPD-223252, PMID: 35754291

[56] Cornelissen VA Fagard RH Coeckelberghs E Vanhees L. Impact of resistance training on blood pressure and other cardiovascular risk factors: a meta-analysis of randomized, controlled trials. Hypertension. (2011) 58:950–8. doi: 10.1161/HYPERTENSIONAHA.111.177071, PMID: 21896934

[57] Babatunde OO Jordan JL Van der Windt DA Hill JC Foster NE Protheroe J. Effective treatment options for musculoskeletal pain in primary care: a systematic overview of current evidence. PLoS One. (2017) 12:e0178621–51. doi: 10.1371/journal.pone.0178621, PMID: 28640822 PMC5480856

[58] Tanasescu M Leitzmann MF Rimm EB Willett WC Stampfer MJ Hu FB. Exercise type and intensity in relation to coronary heart disease in men. JAMA. (2002) 288:1994–2000. doi: 10.1001/jama.288.16.1994 12387651

[59] Gordon BR McDowell CP Hallgren M Meyer JD Lyons M Herring MP. Association of efficacy of resistance exercise training with depressive symptoms: meta-analysis and meta-regression analysis of randomized clinical trials. JAMA Psychiat. (2018) 75:566–76. doi: 10.1001/jamapsychiatry.2018.0572, PMID: 29800984 PMC6137526

