# Supplementary material for: Effects of resistance and balance exercises for athletic ability and quality of life in people with osteoporotic vertebral fracture: Systematic review and meta-analysis of randomized control trials
Source: Front Med (Lausanne). 2023 Mar 9;10:1135063. doi: 10.3389/fmed.2023.1135063 (PMC10033532; doi:10.3389/fmed.2023.1135063)
Supplement: Supplementary file 1 [file Data_Sheet_1.doc]

Search strategy.

**PUBMED**:

#1 (“Exercises”[MeSH Terms] OR “Activities, Physical”[All Fields] OR “Activity, Physical”[All Fields] OR “Physical Activities”[All Fields] OR “Exercise, Physical”[All Fields] OR “Exercises, Physical”[All Fields] OR “Physical Exercise”[All Fields] OR “Physical Exercises”[All Fields] OR “Exercise Training”[All Fields] OR “Training, Exercise”[All Fields] OR “Training, Exercise”[All Fields])

#2 (“Osteoporotic fracture”[MeSH Terms] OR “Fractures, Osteoporotic”[All Fields] OR “Fracture, Osteoporotic”[All Fields])

#3 (“Fracture, Spinal”[MeSH Terms] OR “Fractures, Spinal”[All Fields] OR “Spinal Fracture”[All Fields])

#4 (“Function”[All Fields] OR “Activities of daily living”[All Fields] OR “Functioning”[All Fields])

#5 #1AND #2 AND #3 AND #4

**Web of Science**

#1 TS=Exercises OR Physical Activities OR Physical Exercise OR Physical Exercises OR Exercise Training

#2 TS=Osteoporotic fracture OR Fractures Osteoporotic

#3 TS=Spinal Fracture OR Fractures Spinal

#4 TS=Function OR Activities of daily living

#5 #1 AND #2 AND #3 AND #4

**EMBASE**

#1 *'* Exercises *'/exp OR '* Physical Activities *'/exp OR '* Physical Exercises *'/exp OR '* Exercise Training *'/exp*

*#2 '* Osteoporotic fracture *'/exp OR '* Fractures Osteoporotic *'/exp*

*#3 '* Spinal Fracture *'/exp OR '* Fractures Spinal *'/exp*

*#4 '* Function *'/exp OR '* Activities of daily living *'/exp*

*#5* #1 AND #2 AND #3 AND #4

**Cochrane Library:**

*#1 '* Exercises *'* or *'* Physical Activities *' or* *'* Physical Exercise *' or* *'* Exercise Training *'*

#2 *'* Osteoporotic fracture *' or* *'* Fractures, Osteoporotic*'*

#3 *'* Spinal Fracture *' or* *'* Fracture, Spinal*'*

#4 *'* Function *' or '* Activities of daily living*'*

#5 #1AND #2AND#3AND#4

**CNKI**

(SU=*'* Exercises *' + '* Physical Activities *'*) AND (SU=*'* Osteoporotic fracture *' + '* Fractures Osteoporotic *'*) AND (SU=*'* Function *'+ '* Activities of daily living *'*)

**The forest plot for subgroup analysis by age**


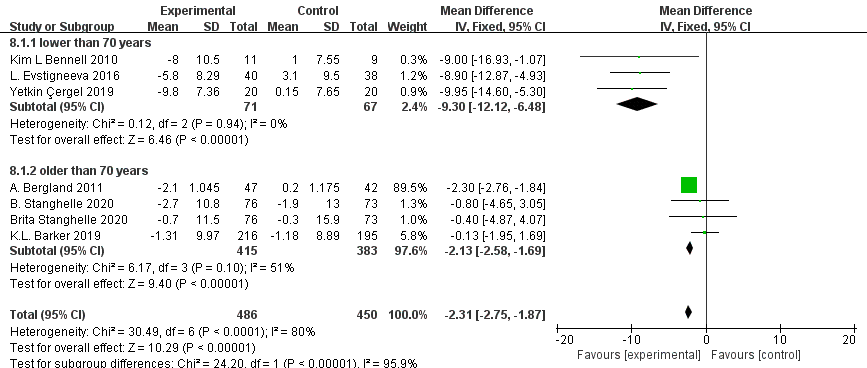


QUALEFFO-41


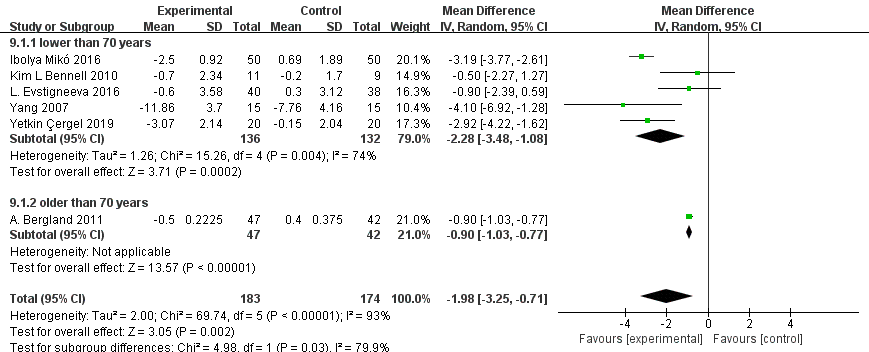


Time Up and Go


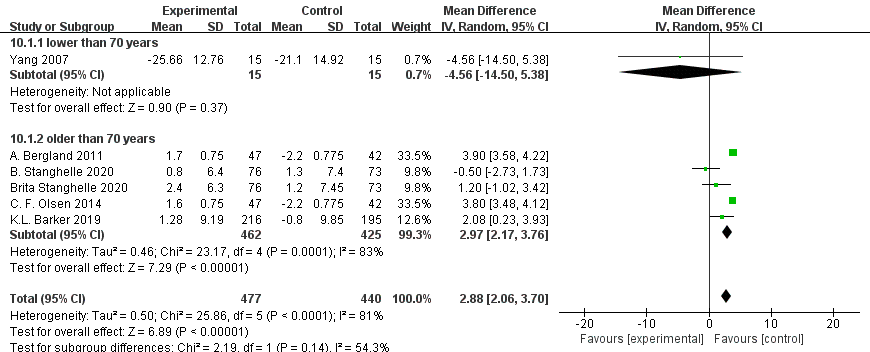


Functional Reach


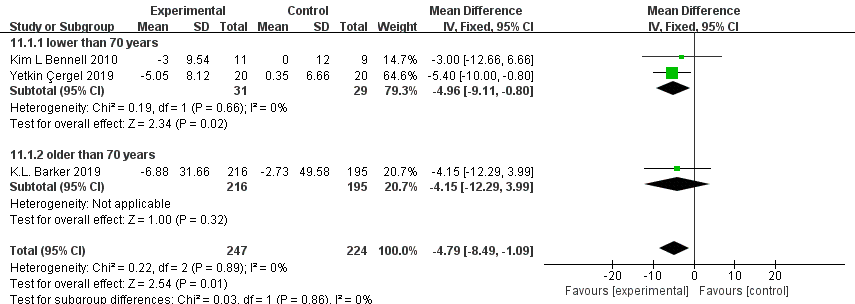


Kyphosis

**The forest plot for subgroup analysis by exercise time**


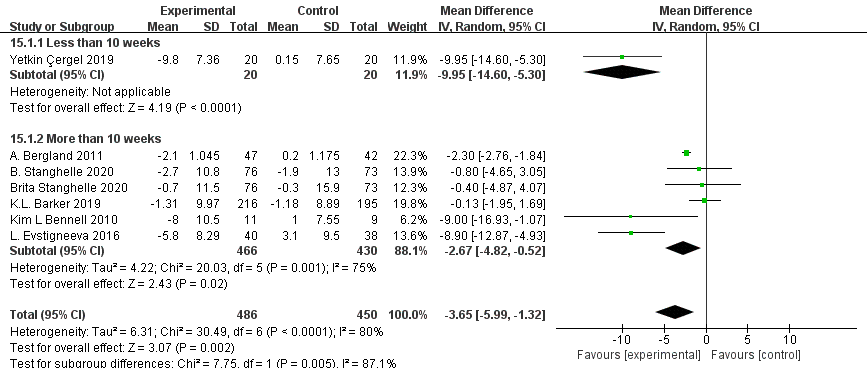


QUALEFFO-41


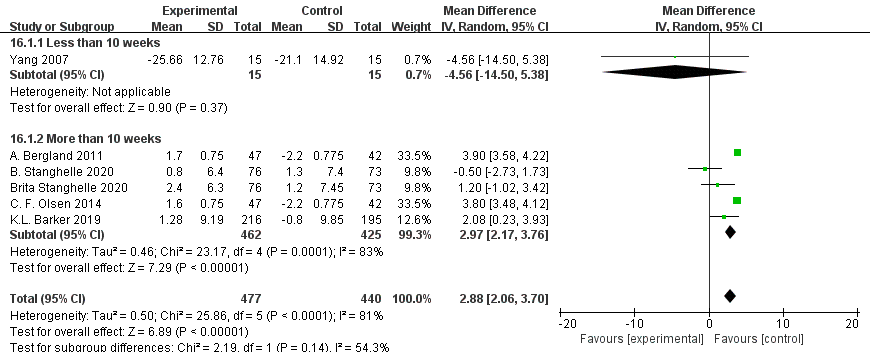


Functional Reach


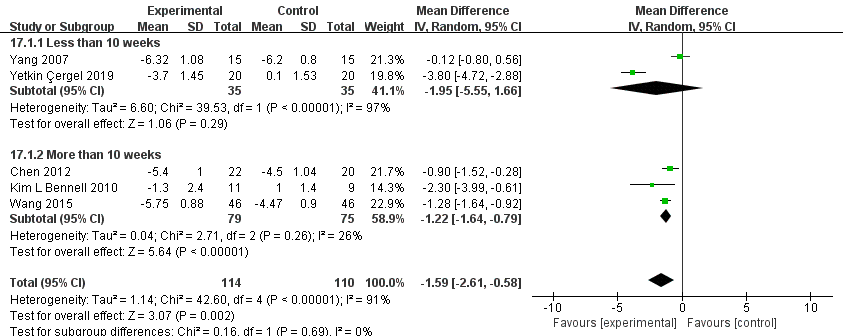


VAS
